# Phase-Amplitude Relations for a Particle with a Superposition of Two Energy Levels in a Double Potential Well

**DOI:** 10.3390/e24030312

**Published:** 2022-02-22

**Authors:** Ofir Flom, Asher Yahalom, Jacob Levitan, Haggai Zilberberg

**Affiliations:** 1 Department of Electrical & Electronic Engineering, Ariel University, Kiriat Hamata P.O. Box 3, Ariel 40700, Israel; ofirshelly7@gmail.com; 2 Center for Astrophysics, Geophysics, and Space Sciences (AGASS), Ariel University, Ariel 40700, Israel; 3 Department of Physics, Ariel University, Kiriat Hamata P.O. Box 3, Ariel 40700, Israel; levitan@ariel.ac.il; 4 Israel Heritage Department, Ariel University, Kiriat Hamata P.O. Box 3, Ariel 40700, Israel; haggaiz@ariel.ac.il

**Keywords:** quantum theory, double potential well, phase-amplitude relations, wave function, conditions of log analytical uncertainty

## Abstract

We study the connection between the phase and the amplitude of the wave function and the conditions under which this relationship exists. For this we use the model of particle in a box. We have shown that the amplitude can be calculated from the phase and vice versa if the log analytical uncertainty relations are satisfied.

## 1. Introduction

In classical physics position and momentum are two conjugate variables that determine motion. It can be said by analogy that amplitude and phase are used in a similar role in quantum mechanics. However, the analogy is not perfect. Pauli [1] asked whether the wave function can be constructed from the knowledge of a set of amplitudes only. Lamb [2] argued that from a set of values of the amplitude of the wave function and their rates of change, the wave function including its phase will be found uniquely. Counter examples were later given [3,4] and now it turns out that the knowledge of amplitude and certain information about the analytical values of the wave function are required together to construct the quantum states. In the study of Guimaraes and Baseia [5] situations with defined phases for stationary or moving type of fields were produced.

Rayleigh [6] showed in the field of classical waves that through the interference between the research wave and the researcher wave, the magnitude and phase of acoustic waves can be determined individually, that is, by finding the minimum and zero values. Mandel and Wolf [7] noted with reference to the ‘complex analytic signal’ (an electromagnetic field with positive frequency components) that the position of the zeros (from which the phase can be determined) and the intensity represent two groups of information intertwined by the analytical value of the wave.

Interference of optical waves is clearly a phase phenomenon. In classical systems this results from the superposition of positive and negative real wave amplitudes. Phase interference can be used in optical systems to obtain a type of quantum measurement, known as non-destructible measurements [8] (chapter 19). This is done to make a measurement that does not change any value of the system at the expense of other values that may change. In optics, the phase is the one that can act as a test to determine the intensity (or number of photons). The phase may change during measurement, while the number of photons does not change [7,9]. To conclude, we will note the theoretical demonstration, presented in [10], which shows that any operator (discrete, finite dimensional) can be constructed using only optical means.

The question of determining the phase of a field (classical or quantum, such as a wave function) from a modulus (absolute value, amplitude) of the field along an actual parameter (for which a single experimental determination is possible), is known as the ‘phase problem’ [7]. The relations derived in the next section represent a formal description for determining the phase given the amplitude, and vice versa.

In 1984 Berry described his discovery of time-independent phase changes in multi-component states known as a geometric/topological phase or Berry phase [11] based, among others, on Aharonov and Bohm who discovered the topologically acquired phase [12] named after them. In their discovery, they showed that when an electron moves along a closed path along which the magnetic field is zero, it acquires an observed phase change that is proportional to the “vector potential”. The topological aspect, i.e., that the path is within a multi-linked part of space (in physical terms, the closed path cannot be contracted without encountering a magnetic field), has also been shown to be of great importance [13,14], mainly through extensions and applications of the phase-change concept of Aharonov–Bohm [15] which led to a number of developments in many fields of physics [16]. The term “open path phase” denotes a fully non-cyclical development [17,18]. This term, unlike the value of the Berry phase, is not a measurable constant, but is accessible, in part, by experiments.

As already noted, the Berry phase and the open path phase indicate changes in the phases of the components of the state, rather than the overall phase change of the wave function, which belongs to the “dynamic phase” [11,19]. In some cases the presence of more than one component in the state function is a topological effect. This determination is based on Longuet–Higgins’ theorem “Topological Test for a Intersections” [20], which states that if a given wave function of an electronic state changes sign when it moves around a loop in a nuclear configuration space, then the state must be degenerate with another state at some point in a loop.

To summarize, regarding the effects of the phase in complex states, we will look at the two ways in which it is possible to arrive at a complex description of a phenomenon that takes place in the real world:

First, the time-dependent wave function is necessarily complex due to the form of the Schrödinger equation that is time-dependent, which includes the square root of minus one *i*. Second, there are also defined functions that do not include the i (such as the Schrödinger equation that is independent of time). Here, too, the wave equation can be complex by having some of the variables obtain complex values. This allows for the removal of possible ambiguities that arise in the solution at a singular point that can be infinite. In addition to this it can often be useful to refer to a number of physical parameters that appear in theory as a complex quantity and that the wave function will include analytical values in relation to them. This formal procedure may even include basic constants such as e,h, and so on.

Yahalom and Englman [21] presented an analytical formulation of a one dimensional scattering process of a microscopic wave packet, which is returned from an infinite potential barrier. It has been found that under conditions suitable for the electron, there is a reciprocal relationship (Kramers–Kronig) between the phase and the amplitude of the wave function of the propagating particle. These interrelationships show the analytical part (phase) uniquely from within the modulus.

The physical basis for this relationship was clarified in [22,23], as stemming from the lower delineation of energies. When the analytical conditions are not fully met, for example, due to being zero points in the wave function, the phase can still be calculated from the amplitude, which is given as a function of the composite time with the introduction of the conditions of Toll [24]. When the underlying analytical conditions are fully met, then they are sufficient to calculate the amplitude as a function of time, i.e., along the actual t-axis.

Following Yahalom and Englman’s research, the present study will seek to expand their analysis and present a reciprocal relationship between the modulus-log and the phase of superposition of the two energy levels of the particle in an infinite potential well at the center of which there is a finite barrier. We remark that the boundary conditions in a well are different, however, than the boundary conditions in the case of an infinite barrier described in [21].

## 2. The Wave Function

The wave function for a time independent single dimensional Hamiltonian is given by the equation:

(1) $$\Psi \left(x,t\right)=\sum_{n=0}^{\infty }A_{n}\psi_{n}\left(x\right)e^{-i\frac{E_{n}}{\hbar }t}$$

When $\psi_{n}\left(x\right)$ are the eigenfunction of the Hamiltonian, $E_{n}$ are the eigenenergies of the Hamiltonian, *ℏ* is a plank constant divided by 2π, *x* and *t* are space and time, respectively, $A_{n}$ are coefficients that express the placement of different energy levels. The normal wave function satisfies:

(2) $$\int_{-\infty }^{\infty }{\left|\Psi \left(x,t\right)\right|}^{2}dx=1$$

## 3. Temporal Kramers–Kronig Relations Theory

The wave function is a complex function and therefore the following relationship is satisfied:

(3) $$\ln\left[\Psi \left(x,t\right)\right]=\ln\left|\Psi \left(x,t\right)\right|+i\phi \left(x,t\right)$$

where |Ψ(x,t)| is the amplitude and ϕ(x,t)=arg[Ψ(x,t)] is the phase. If proper analytical conditions are met the phase and amplitude are related by the temporal Kramers–Kronig relations, which are reminiscent in form with the relations obtained by Kramers and Kronig for the real and imaginary parts of the dielectric function [25]. However, there are also differences, in particular the classical Kramers–Kronig relations are derived in the complex frequency plane, while our results are derived in the complex time plane. To avoid confusion, we use the term **temporal** Kramers–Kronig relations whenever it is appropriate. Another major difference is that we deal with the quantum wave function and not with the electromagnetic dielectric function as was done originally by Kramers and Kronig, hence the physical object under consideration is entirely different. An analysis of ln[Ψ(x,t)] in the lower half of the *t*-plane is required for using the Cauchy theorem to link the real and the imaginary parts of ln[Ψ(x,t)] on the real axis. For all complex times *z* within a closed contour *C* in the lower half of the *t*-plane, Cauchy theorem gives:

(4) $$\ln\left[\Psi \left(x,z\right)\right]=\frac{1}{2\pi i}\oint_{C}\frac{\ln\left[\Psi \left(x,t^{\prime }\right)\right]}{t^{\prime }-z}dt^{\prime }$$

Provided that ln[Ψ(x,t)] is analytical inside the contour.

We assume that the contour *C* can be chosen to include the real t-axis and a large infinite semicircle in the lower half of the plane. In case the logarithm of the wave function vanishes on the circle half at infinity, the Cauchy integral can be written as follows:

(5) $$\ln\left[\Psi \left(x,z\right)\right]=\frac{1}{2\pi i}\int_{-\infty }^{\infty }\frac{\ln\left[\Psi \left(x,t^{\prime }\right)\right]}{t^{\prime }-z}dt^{\prime }$$

Taking the limit as a complex time approaching the real axis from below, we write z=t+iδ in (5):

(6) $$\ln\left[\Psi \left(x,t+i\delta \right)\right]=\frac{1}{2\pi i}\int_{-\infty }^{\infty }\frac{\ln\left[\Psi \left(x,t^{\prime }\right)\right]}{t^{\prime }-t-i\delta }dt^{\prime }$$

**Figure 1:**
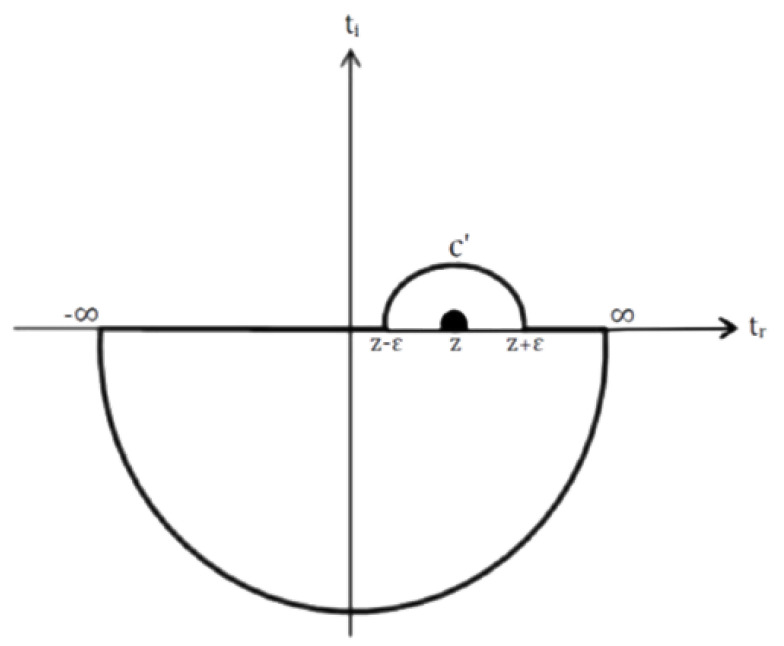
The lower half of the complex time plane.

Taking the limit δ→0 we may contain the point inside the trajectory by drawing a tiny semicircle over point *t* (see Figure 1). The denominator can be formally written as:

(7) $$\frac{1}{t^{\prime }-t-i\delta }=P\left(\frac{1}{t^{\prime }-t}\right)+\pi i\delta \left(t^{\prime }-t\right)$$

where *P* is the principal value. The delta function is used to describe the contribution from the small semicircle that goes in against the clock direction over the pole at $t^{\prime }=t$. Using (5) and simply rearranging turns (6) into:

(8) $$\ln\left[\Psi \left(x,t\right)\right]=\frac{1}{\pi i}P\int_{-\infty }^{\infty }\frac{\ln\left[\Psi \left(x,t^{\prime }\right)\right]}{t^{\prime }-t}dt^{\prime }$$

The real and imaginary parts of this equation are:

(9) $$\begin{array}{c} \ln\left|\Psi \left(x,t\right)\right|=\frac{1}{\pi }P\int_{-\infty }^{\infty }\frac{\phi \left(x,t\right)}{t^{\prime }-t}dt^{\prime }\\ \phi \left(x,t\right)=-\frac{1}{\pi }P\int_{-\infty }^{\infty }\frac{\ln\left|\Psi \left(x,t\right)\right|}{t^{\prime }-t}dt^{\prime } \end{array}$$

The temporal Kramers–Kronig relations expresses the relation between the real part and the imaginary part of the logarithm of the wave function. As stated, the relation expressed in Equation (9) is true only in the case where the function is log-analytic in the lower half of the plane. This means that the function will have neither singular points nor zeros in the lower half of the plane.

## 4. Checking Zeros and Singular Points in the Lower Half of the Complex Time

Our wave function (the general solution for the Schrödinger equation that is time-independent) is given in formula (1) where $\omega_{n}=\frac{E_{n}}{\hbar }$ and also holds:

(10) $$\omega_{1}\leq \omega_{2}\leq \omega_{3}\leq \cdot \cdot \cdot \leq \omega_{n}\leq \cdot \cdot \cdot$$

We want to test analyticity in the lower half of the complex time $t=t_{r}+it_{i}$ so we will write:

(11) $$e^{-i\omega_{n}t}=e^{-i\omega_{n}\left(t_{r}+it_{i}\right)}=e^{-i\omega_{n}t_{r}}e^{\omega_{n}t_{i}}$$

We will write the function (1) as follows:

(12) $$\begin{array}{ccc} \Psi \left(x,t\right) & = & e^{-i\omega_{1}t}\sum_{n}A_{n}\psi_{n}\left(x\right)e^{-i\left(\omega_{n}-\omega_{1}\right)t}\\ & = & e^{-i\omega_{1}t_{r}}e^{\omega_{1}t_{i}}\sum_{n}A_{n}\psi_{n}\left(x\right)e^{-i\left(\omega_{n}-\omega_{1}\right)t_{r}}e^{\left(\omega_{n}-\omega_{1}\right)t_{i}} \end{array}$$

When $\Delta \omega_{n}\equiv \omega_{n}-\omega_{1}\geq 0$, it is clear that the function has no singular points in the lower half of the plane and therefore we will concentrate on identifying the zeros if any. We will define:

(13) $$\tilde{\Psi }\left(x,t\right)=\sum_{n}A_{n}\psi_{n}\left(x\right)e^{-i\left(\omega_{n}-\omega_{1}\right)t}=\sum_{n}A_{n}\psi_{n}\left(x\right)e^{-i\Delta \omega_{n}t}=\sum_{n}A_{n}\psi_{n}\left(x\right)e^{-i\Delta \omega_{n}t_{r}}e^{\Delta \omega_{n}t_{i}}$$

We will calculate a limit of Ψ(x,t) when the imaginary part of time approaches to minus infinity:

(14) $$\Psi_{\infty }\left(x,t\right)\equiv \lim_{t_{i}\to -\infty }\Psi \left(x,t\right)=A_{1}\psi_{1}\left(x\right)e^{-i\omega_{1}t}$$

We will now define an asymptotic wave function:

(15) $$\hat{\Psi }\left(x,t\right)=\frac{\Psi \left(x,t\right)}{\Psi_{\infty }\left(x,t\right)}=\frac{\sum_{n}A_{n}\psi_{n}\left(x\right)e^{-i\omega_{n}t}}{A_{1}\psi_{1}\left(x\right)e^{-i\omega_{1}t}}=\sum_{n}\left(\frac{A_{n}\psi_{n}\left(x\right)}{A_{1}\psi_{1}\left(x\right)}\right)e^{-i\Delta \omega_{n}t}$$

We need a log analytic function, i.e., a function that does not accept values of zero or infinity in the lower half of the complex time plane. Moreover, it is required that the function approaches unity on the infinity circle so that the logarithm vanishes on this circle. Thus we will use the asymptotic function. We have not yet proved that this function is log analytical and this can only be proved with respect to individual cases where the temporal Kramers–Kronig relations exists. We will first deal with the superposition of two eigenfunction

(16) $$\hat{\Psi }\left(x,t\right)=1+\frac{A_{2}}{A_{1}}\frac{\psi_{2}}{\psi_{1}}e^{-i\Delta \omega t}$$

When $\Delta \omega =\omega_{2}-\omega_{1}>0$. In order for the wave function to be log analytical so that $\ln\left[\Psi \left(x,t\right)\right]$ is not diverging at any point, we must find the condition under which there are no zeros in the complex plane of time. This is a prerequisite for the temporal Kramers–Kronig relations to be satisfied. That is, since $\left|e^{-i\Delta \omega t}\right|<1$ then we require that $\left|\frac{A_{2}}{A_{1}}\frac{\psi_{2}}{\psi_{1}}\right|<1$. Assuming that the placement coefficients are equal $A_{2}≅A_{1}$ the condition for the absence of zeros is:

(17) $$\left|\frac{\psi_{2}}{\psi_{1}}\right|<1.$$

Therefore if condition (17) is met $\left|\hat{\Psi }\left(x,t\right)\right|>0$ at each and every point in the lower part of the complex plane.

## 5. The Principle of Log Analytical Uncertainty

The eigenfunctions can be written as follows:

(18) $$\psi_{1}\left(x\right)=\psi \left(x,E_{1}\right),\;\psi_{2}\left(x\right)=\psi \left(x,E_{2}\right).$$

where $E_{2}=E_{1}+\Delta E$ and ΔE is the difference in eigenenergies levels. Thus we can write to first order in ΔE:

(19) $$\psi_{2}\left(x\right)=\psi \left(x,E_{2}\right)=\psi \left(x,E_{1}+\Delta E\right)=\psi \left(x,E_{1}\right)+\Delta E\frac{d\psi_{1}}{dE_{1}}$$

Then following Equation (19) we have:

(20) $$\left|\frac{\psi_{1}+\Delta E\frac{d\psi_{1}}{dE_{1}}}{\psi_{1}}\right|=\left|1+\frac{\Delta E\frac{d\psi_{1}}{dE_{1}}}{\psi_{1}}\right|<1\;\;$$

Hence:

(21) $$\sqrt{\left(1+\frac{\Delta E\frac{d\psi_{1}}{dE_{1}}}{\psi_{1}}\right)\left(1+\frac{\Delta E\frac{d\psi_{1}^{*}}{dE_{1}}}{\psi_{1}^{*}}\right)}<1$$

This can be written to first order approximation as:

(22) $$\sqrt{\left[1+\Delta E\left(\frac{\frac{d\psi_{1}}{dE_{1}}}{\psi_{1}}+\frac{\frac{d\psi_{1}^{*}}{dE_{1}}}{\psi_{1}^{*}}\right)\right]}<1$$

By using Taylor first order approximation $\sqrt{1+x}≃1+\frac{x}{2}$ we may write:

(23) $$1+\frac{\Delta E}{2}\left(\frac{\frac{d\psi_{1}}{dE_{1}}}{\psi_{1}}+\frac{\frac{d\psi_{1}^{*}}{dE_{1}}}{\psi_{1}^{*}}\right)<1$$

and obtain:

(24) $$\Delta ERe\left(\frac{\frac{d\psi_{1}}{dE_{1}}}{\psi_{1}}\right)<0$$

(25) $$\Delta ERe\left(\frac{d\ln\psi_{1}}{dE_{1}}\right)<0$$

Since the energy difference must be positive we obtain:

(26) $$Re\left(\frac{d\ln\psi_{1}}{dE_{1}}\right)<0$$

(27) $$\frac{d\ln\left|\psi_{1}\right|}{dE_{1}}<0$$

Simplify the above according to the chain rule:

(28) $$\frac{d\ln\left|\psi_{1}\right|}{d\left|\psi_{1}\right|}\frac{d\left|\psi_{1}\right|}{dE_{1}}<0$$

(29) $$\frac{1}{\left|\psi_{1}\right|}\frac{d\left|\psi_{1}\right|}{dE_{1}}<0$$

Since the amplitude of the function is positive it follows that:

(30) $$\frac{d\left|\psi_{1}\right|}{dE_{1}}<0$$

We will test Equation (30) in a specific model which is the Double Potential Well Model to be described below.

## 6. Combining a Double Potential Well Model

**Figure 2:**
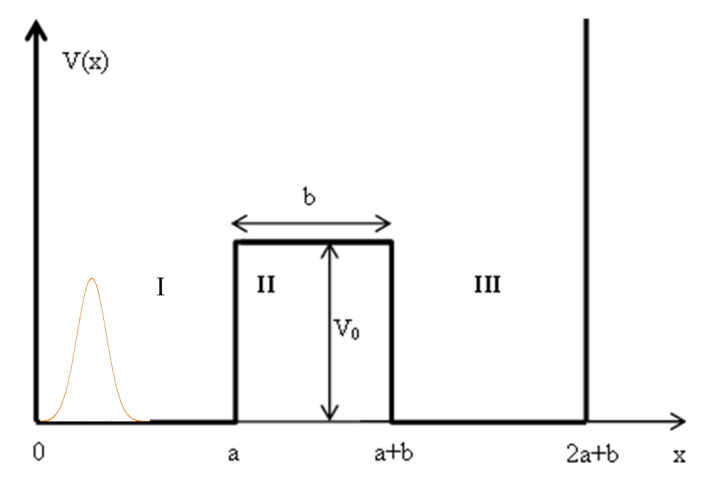
Symmetrical Double Potential Well.

We consider a potential well as depicted in Figure 2. A particle is moving to the right from the left hand side of a finite-sized barrier located in the center of the well at x=a. The wave function of the particle is represented as the superposition of the two energy levels of the particle in a double well. Quantum mechanics predicts that the particle will be returned after encountering the barrier with high probability. However there is also a probability of tunneling through the barrier and towards the right side of the potential well as shown in Figure 2:

The eigenfunctions of the double well are given in Equation (31):

(31) $$\psi \left(x,E\right)=A\left\{\begin{array}{c} \sin\left(kx\right),\;0<x\leq a\\ \sin\left(ka\right)\cosh\left[q\left(x-a\right)\right]+\frac{k}{q}\cos\left(ka\right)\sinh\left[q\left(x-a\right)\right],\;a<x\leq a+b\\ \cos\left[k\left(x-a-b\right)\right]\left[\cosh\left(qb\right)\sin\left(ka\right)+\frac{k}{q}\sinh\left(qb\right)\cos\left(ka\right)\right]\\ +\sin\left[k\left(x-a-b\right)\right]\left[\cosh\left(qb\right)\cos\left(ka\right)+\frac{q}{k}\sinh\left(qb\right)\sin\left(ka\right)\right],\\ \;a+b<x\leq 2a+b \end{array}\right.$$

When $k=\sqrt{\frac{2m}{\hbar^{2}}E}$ and $q=\sqrt{\frac{2m}{\hbar^{2}}\left(V_{0}-E\right)}$. The expression for energy as a function of the wave-number *k* is given in the formula

(32) $$E_{n}=\frac{\hbar^{2}k_{n}^{2}}{2m}$$

and will be related to *q* according to equation:

(33) $$E_{n}=V_{0}+\frac{\hbar^{2}q_{n}^{2}}{2m}$$

Following Equation (30) and using the chain rule we obtain the following inequality as a condition for the Kramers Kronig relations to hold:

(34) $$\frac{d\left|\psi_{1}\right|}{dk_{1}}\frac{dk_{1}}{dE_{1}}<0$$

Since the derivative $\frac{dk_{1}}{dE_{1}}>0$ is positive it will not affect the derivative of the wave function and therefore we obtain the condition:

(35) $$\frac{d\left|\psi_{1}\right|}{dk_{1}}<0$$

In order to establish the domain without zero points we will use Equation (35). In the domain left to the barrier we have according to Equation (31):

(36) $$\frac{d\left|\psi_{11}\right|}{dk_{1}}=\frac{d\sin\left(k_{1}x\right)}{dk_{1}}=x\cos\left(k_{1}x\right)<0$$

Since *x* is positive then we are left with the cosine function which is negative provided that: $\frac{\pi }{2}<k_{1}x<\frac{3\pi }{2}$, we will note that it is not possible to reach the upper limit and therefore it is irrelevant. The momentum is given by the relation:

(37) p=ℏk.

Therefore the result is:

(38a) $$\begin{array}{c} p_{1}x>\frac{\hbar \pi }{2} \end{array}$$

or

(38b) $$p_{1}x>\frac{h}{4}$$

If those condition are met, the function is a log analytical and satisfies the temporal Kramers–Kronig relations, meaning that the amplitude can be calculated using the phase and the phase can be calculated by using the amplitude. When the condition is not met it is not possible to calculate the phase from the amplitude and the amplitude from the phase, so we have uncertainty about the second magnitude given the first magnitude. For this reason the above inequality, is denoted the principle of log analytical certainty. Thus, it can also be said that there is uncertainty in the relationship between the phase and amplitude in the field in which the following condition is met:

(39a) $$\begin{array}{c} p_{1}x<\frac{\hbar \pi }{2} \end{array}$$

or

(39b) $$p_{1}x<\frac{h}{4}$$

**Figure 3:**
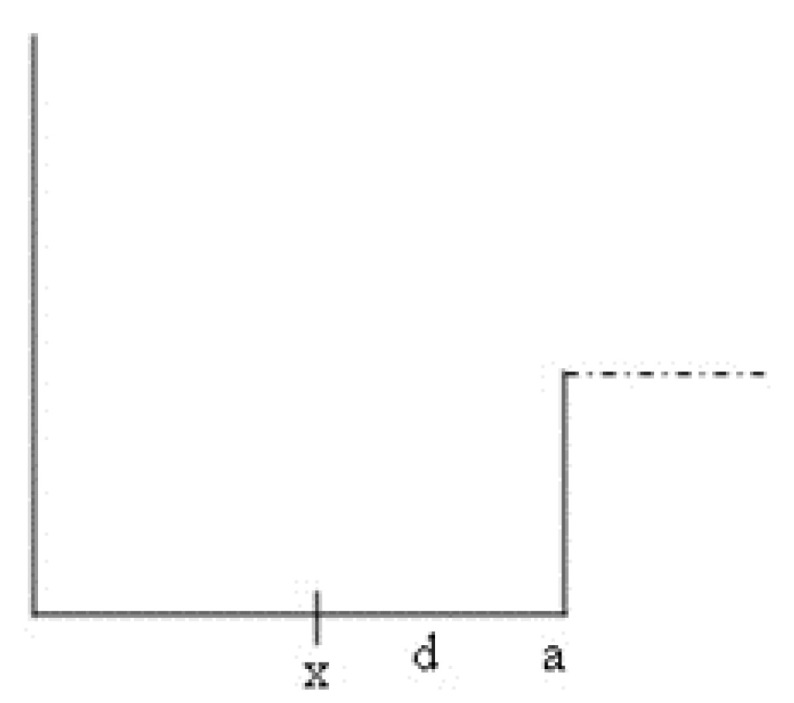
Distance between the measuring point and the barrier.

According to inequalities (39a) and (39b) the larger the momentum (or energy) of the particle, the smaller the x area where the uncertainty between the phase and the amplitude takes place, and the greater the area where certainty exists. Things can also be presented differently if we look at the distance *d* between the barrier and the measuring point (see Figure 3):

(40a) $$\begin{array}{c} d=a-x \end{array}$$

(40b) x=a−d

(40c) $$p_{1}\left(a-d\right)<\frac{h}{4}$$

(40d) $$p_{1}a-p_{1}d<\frac{h}{4}$$

(40e) $$p_{1}a-\frac{h}{4}<p_{1}d$$

(41) $$d>a-\frac{h}{4p_{1}}$$

This condition means that as *p* increases (i.e., as energies increase) so, the area of certainty adjacent to the barrier increases and then the area of uncertainty decreases. It is also seen that as the width of the well increases, *d* (the range of certainty) increases. We will multiply the two sides of inequality (41) by $p_{1}$ and use Equation (37) to obtain:

(42) $$p_{1}d>\hbar k_{1}a-\frac{h}{4}=h\left(\frac{k_{1}a}{2\pi }-\frac{1}{4}\right)$$

**Figure 4:**
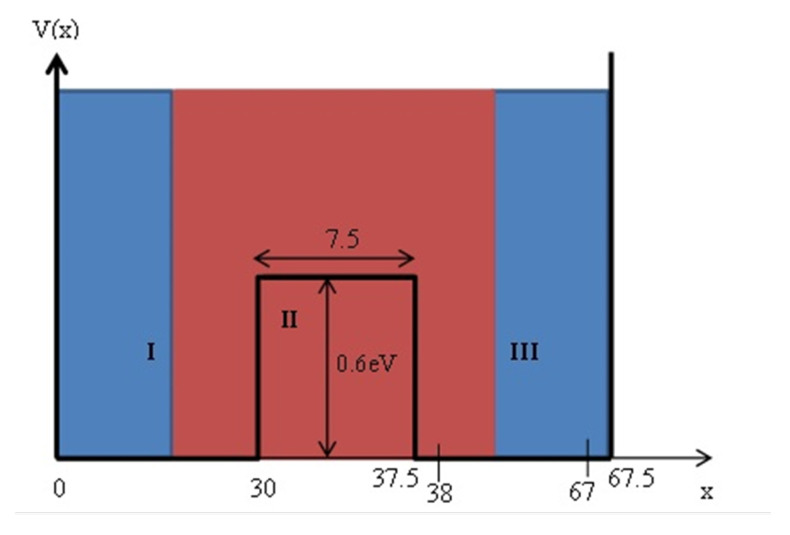
The double well with specific well dimensions, areas of certainty are colored in red and uncertainty in blue.

The $k_{1}a$ product is constant under single potential well conditions (without a barrier), whereas in the case of a double well it increases moderately with the increase in *a* (for example, with doubling *a* the product increases by 4.55%, and by taking four time of *a* times the product increases by 6.95%). The above inequality is reminiscent in its form of Heisenberg’s principle of uncertainty $\Delta p\Delta x\geq \frac{\hbar }{2}$. It can be seen, however, that Heisenberg’s inequality does not define a range in which the certainty or uncertainty exists, but rather defines a minimum magnitude to which the product of standard deviations of momentum and position of the particle wave function is equal. So as the standard deviation of the momentum increases so we will talk about a smaller initial standard deviation of the position, whereas in the present case inequality describes the condition for being in an area where there is an uncertainty in the relationship between phase and amplitude. We see that near the barrier we have certainty in the sense that the amplitude and phase can be reconstructed from each other while at a distance exceeding the value derived the phase can not be reconstructed from the amplitude and vice versa. In the domain within the barrier (the second domain) it can be seen according to Figure 4 that there is no uncertainty since the derivative will always be negative. That is, at each point there is a phase-amplitude relationship.

## 7. Areas of Certainty and Uncertainty beyond the Barrier

The expression for the energy eigenfunction in the third domain (the domain to the right of the barrier) is:

(43) $$\begin{array}{c} \begin{array}{c} \psi_{3,1}\left(x\right)=\cos\left[k_{1}\left(x-a-b\right)\right]\left[\cosh\left(q_{1}b\right)\sin\left(k_{1}a\right)+\frac{k_{1}}{q_{1}}\sinh\left(q_{1}b\right)\cos\left(k_{1}a\right)\right]\\ +\sin\left[k_{1}\left(x-a-b\right)\right]\left[\cosh\left(q_{1}b\right)\cos\left(k_{1}a\right)+\frac{q_{1}}{k_{1}}\sinh\left(q_{1}b\right)\sin\left(k_{1}a\right)\right] \end{array} \end{array}$$

We will define $x^{\prime }=x-a-b$ and simplify the function as follows:

(44) $$\psi_{3,1}\left(x,E\right)=a_{1}\cos\left(k_{1}x^{\prime }\right)+b_{1}\sin\left(k_{1}x^{\prime }\right)$$

where:

(45) $$a_{1}=\cosh\left(q_{1}b\right)\sin\left(k_{1}a\right)+\frac{k_{1}}{q_{1}}\sinh\left(q_{1}b\right)\cos\left(k_{1}a\right)$$

(46) $$b_{1}=\cosh\left(q_{1}b\right)\cos\left(k_{1}a\right)+\frac{q_{1}}{k_{1}}\sinh\left(q_{1}b\right)\sin\left(k_{1}a\right)$$

Taking a derivative of the function in Equation (44) by Equation (35):

(47) $$\begin{array}{c} \begin{array}{c} \frac{d\psi_{3,1}}{dk_{1}}=\frac{da_{1}}{dk_{1}}\cos\left(k_{1}x^{\prime }\right)-x^{\prime }a_{1}\sin\left(k_{1}x^{\prime }\right)+\frac{db_{1}}{dk_{1}}\sin\left(k_{1}x^{\prime }\right)+x^{\prime }b_{1}\cos\left(k_{1}x^{\prime }\right)\\ =\left(\frac{db_{1}}{dk_{1}}-x^{\prime }a_{1}\right)\sin\left(k_{1}x^{\prime }\right)+\left(\frac{da_{1}}{dk_{1}}+x^{\prime }b_{1}\right)\cos\left(k_{1}x^{\prime }\right)\\ =a_{2}\sin\left(k_{1}x^{\prime }\right)+b_{2}\cos\left(k_{1}x^{\prime }\right) \end{array} \end{array}$$

When:

(48) $$a_{2}=\frac{db_{1}}{dk_{1}}-x^{\prime }a_{1}$$

(49) $$b_{2}=\frac{da_{1}}{dk_{1}}+x^{\prime }b_{1}$$

In Equations (48) and (49) we will neglect the terms $x^{\prime }b_{1}$ and $x^{\prime }a_{1}$. This can be justified on the basis that the domain of certainty is close the barrier. It is obvious that for a very small $x^{\prime }$ (very close the barrier) the negligence of $x^{\prime }b_{1}$ and $x^{\prime }a_{1}$ is justified. For slightly larger $x^{\prime }$ we notice that the first term in Equation (48) which is $\frac{db_{1}}{dk_{1}}∼a\;b_{1}$ if we assume that *a* and *b* are the same order of magnitude. Now, if $a_{1}$ and $b_{1}$ are the same order of magnitude it is clear that if $x^{\prime }$ is smaller than *a* (or *b*) even if not extremely small the terms $x^{\prime }b_{1}$ and $x^{\prime }a_{1}$ can be neglected in comparison to $\frac{da_{1}}{dk_{1}}$ and $\frac{db_{1}}{dk_{1}}$, respectively. We examined a number of concrete cases and saw that the neglection is indeed justified. As the log analytic condition requires that:

(50) $$\frac{d\psi_{3,1}}{dk_{1}}<0\;\Rightarrow \;a_{2}\sin\left(k_{1}x^{\prime }\right)+b_{2}\cos\left(k_{1}x^{\prime }\right)<0$$

Dividing by a positive cosine and a positive $b_{2}$ the following inequality is obtained as a condition for log analyticity:

(51) $$\tan\left(k_{1}x^{\prime }\right)<-\frac{a_{2}}{b_{2}}$$

Note that the cosine is negative when $x>\frac{\pi }{2k_{1}}$. It can be seen that the log analytical principle does not hold and this is because inequality is reversed. Thus:

(52) $$k_{1}x^{\prime }<\tan^{-1}\left(-\frac{a_{2}}{b_{2}}\right)$$

We will denote:

(53) $$F_{1}\left(k\right)\equiv \tan^{-1}\left(-\frac{a_{2}}{b_{2}}\right)$$

Therefore, the principle of certainty in the third domain of the well is:

(54) $$p_{1}x^{\prime }<\hbar F_{1}\left(k\right)=\frac{h}{2\pi }F_{1}\left(k\right)$$

The condition for uncertainty involves reversing the sign of inequality:

(55) $$p_{1}x^{\prime }>\hbar F_{1}\left(k\right)=\frac{h}{2\pi }F_{1}\left(k\right)$$

It can be said that these inequalities are similar to Heisenberg’s uncertainty principle. From this it can be deduced that in the third domain that provided that the higher the eigenenergies the larger the value on the inequality right side. It will also follow that under the same conditions the area of certainty increases and therefore the area of uncertainty decreases.

A concrete example is given in Figure 4. Where we illustrate that the areas of certainty and uncertainty as derived from our numerical calculations. The following parameters of the double well are assumed: Depth $V_{0}=0.6$ eV, width of each of the two wells a=30 Å, and barrier width b=7.5 Å.

## 8. Finding the Phase-Amplitude Relationship

**Figure 5:**
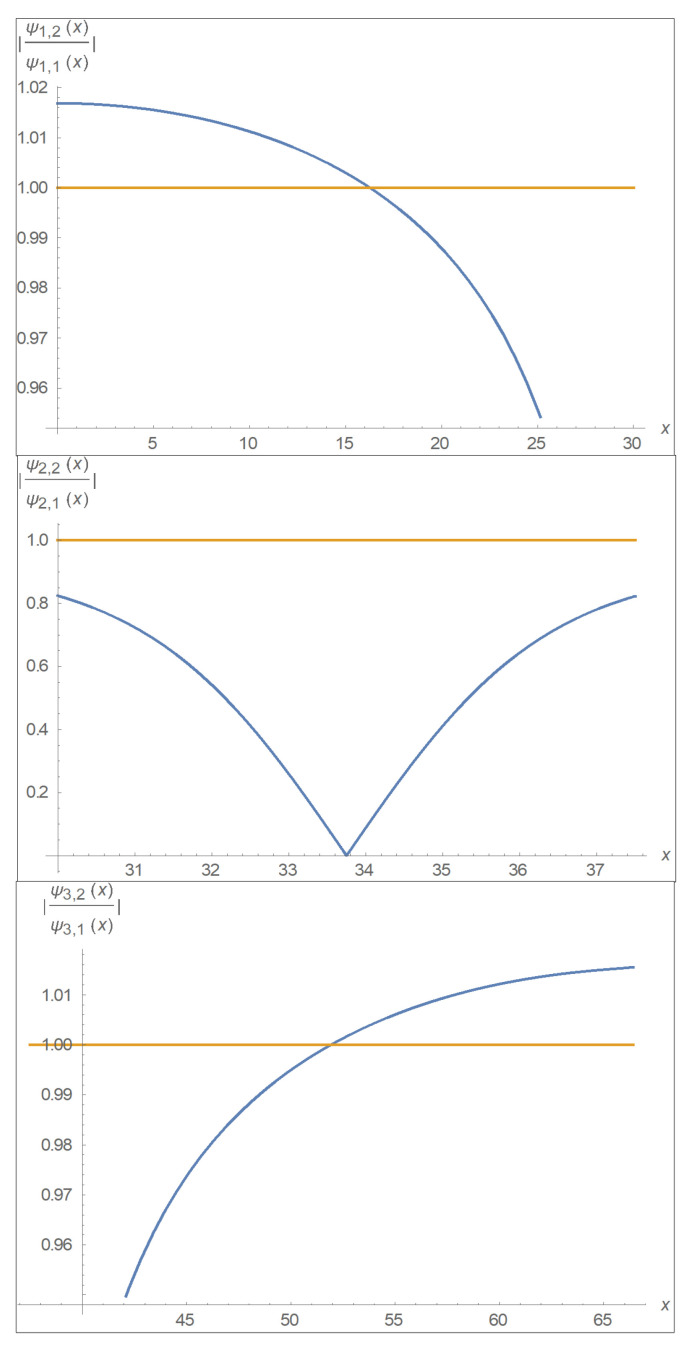
Quotient of the absolute eigen functions in the double well. The top graph depicts the first (left) domain of the well. The middle graph depicts the second domain (inside the barrier) and the lower graph depicts the third domain (right). In each graph one can see where the temporal Kramers–Kronig relations are valid and it is obvious that on both sides of the well the temporal Kramers–Kronig relations takes place only in the vicinity of the barrier, while inside the barrier they take place at all points. For any value of the absolute quotient which is greater than 1 there is uncertainty about the relations between phase and amplitude in the sense that they cannot be deduced from each other.

Once we have identified the certainty area, we will also check numerically, for the sake of demonstration specific conditions. We will use Equation (15) to make an temporal Kramers–Kronig integral and thus test the relationship between phase and amplitude in the following equations:

(56) $$\ln\left|\hat{\psi }\left(x,t\right)\right|=\frac{1}{\pi }P\int_{-\infty }^{\infty }\frac{arg\left[\hat{\psi }\left(x,t^{\prime }\right)\right]}{t^{\prime }-t}dt^{\prime }$$

(57) $$arg\left[\hat{\psi }\left(x,t^{\prime }\right)\right]=-\frac{1}{\pi }P\int_{-\infty }^{\infty }\frac{\ln\left|\hat{\psi }\left(x,t^{\prime }\right)\right|}{t^{\prime }-t}dt^{\prime }$$

where *P* denotes the principal value. Now let’s take the first two eigenenergies levels assuming the parameters: $V_{0}$ = 0.6 eV, a=30 Å, b=7.5 Å, $m=m_{e}=0.5109906\times 10^{6}\;\mathrm{eV}/c^{2}$, $\hbar =6.582122\times 10^{-16}\;\mathrm{eV}\times s$, $c=2.99792458\times 10^{18}$ Å/s. For such conditions the lowest energy levels are $E_{1}=0.034886$ eV and $E_{2}=0.036074$ eV, we also assume that the placement of those levels are equal (at room temperature). We also take into account that the wave numbers are $k_{n}=\sqrt{\frac{2m}{\hbar }E_{n}}$ and $q_{n}=\sqrt{\frac{2m}{\hbar }\left(V_{0}-E_{n}\right)}$. Figure 5 shows the quotient of the absolute eigen functions in each domain of the double well (domains are depicted in Figure 4) and according to the parameters given above, in which it is possible to see where there are no zeros in the complex time plane as the amplitude of the quotient is smaller than one. Throughout the double well it can be seen that phase-amplitude temporal Kramers–Kronig correspondence is expected to take place exclusively in the vicinity of the barrier (i.e., left and right sides close to the barrier and also inside the barrier).

## 9. Testing Phase-Amplitude Relations

We will now examine whether the temporal Kramers–Kronig relations hold. We will look at the first domain of the well and consider the uncertainty relation described in the Equation (39). Let us take a point closest to the barrier (because this is the domain where the phase-amplitude relations should be satisfied), for example, x=25 Å. We will use Equation (56) and write the principal value explicitly:

(58) $$\ln\left|\hat{\psi }\left(x,t\right)\right|=\frac{1}{\pi }\left[\int_{-\infty }^{t-\varepsilon }\frac{arg\left[\hat{\psi }\left(x,t^{\prime }\right)\right]}{t^{\prime }-t}dt^{\prime }+\int_{t+\varepsilon }^{\infty }\frac{arg\left[\hat{\psi }\left(x,t^{\prime }\right)\right]}{t^{\prime }-t}dt^{\prime }\right]$$

**Figure 6:**
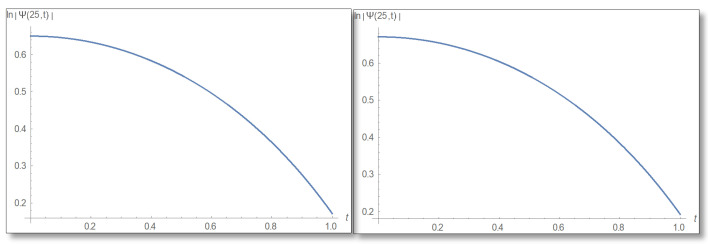
The Graph of the amplitude logarithm in the first domain as a function of time for x=25. The duration 0–1 is depicted. The figure on the left depicts the straight forward calculation, which can be seen to be identical to the temporal Kramers–Kronig integral presented in the right.

**Figure 7:**
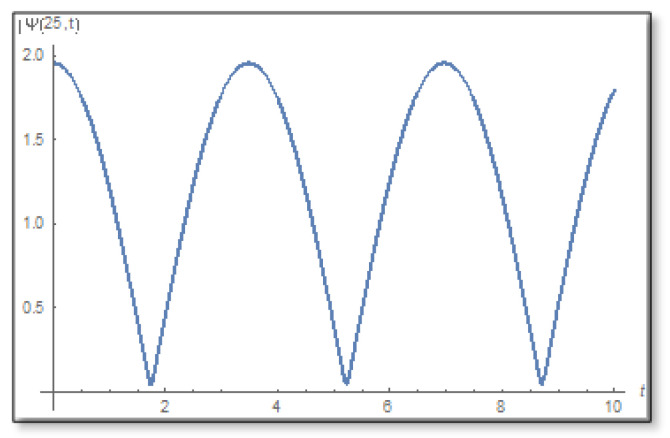
The graph of the amplitude in the first domain as a function of time. This is a graph for x=25 where you can see that the amplitude has no zeros on the time line and also no zeros under the time line. The duration is 0–10.

$\varepsilon =10^{-3}$ is selected. We compare a graph for the amplitude logarithm derived by a standard straight forward calculation to the temporal Kramers–Kronig integral of the phase we obtain that the graphs are the same as can easily be verified in Figure 6. The graph of the amplitude for a longer period of time is shown in Figure 7.

**Figure 8:**
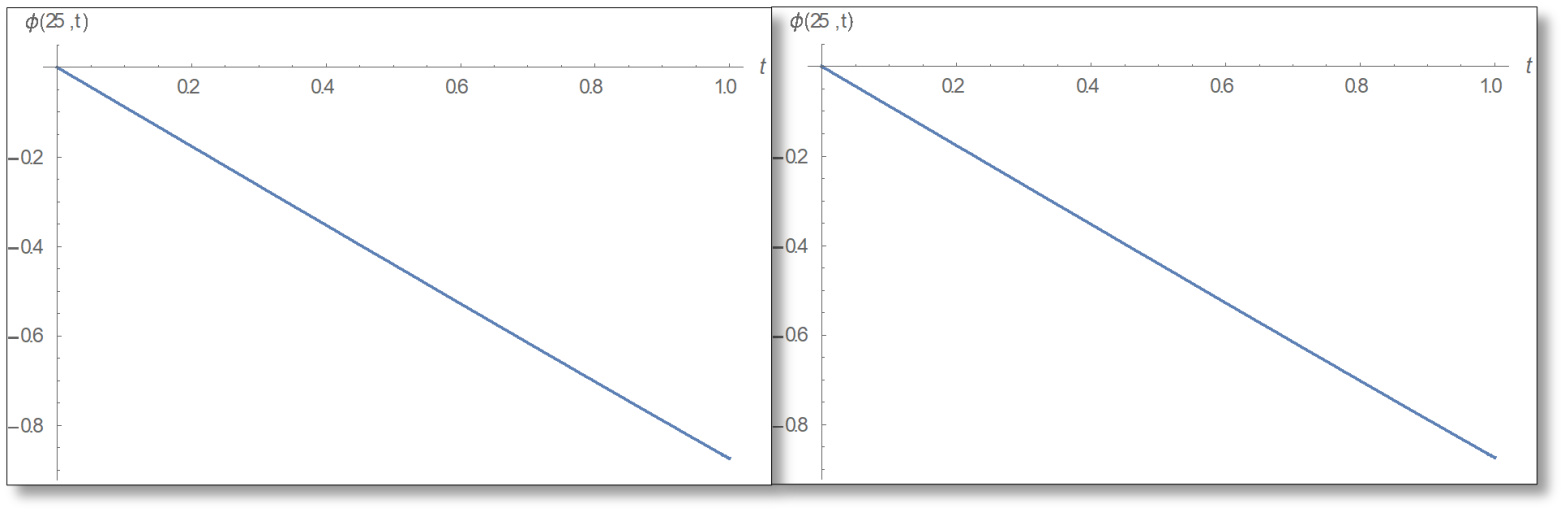
A phase graph in the first domain as a function of time for x=25 for the duration 0–1. On the left the direct evaluation is depicted and on the right is the temporal Kramers–Kronig integral. The two graphs are identical so the mathematical analysis is valid.

Now we will do an Inverse transform to find the phase according to Equation (57) and use the principal value as we did in Equation (58), then we will draw a phase graph as a function of time for straight forward phase calculation and for the temporal Kramers–Kronig calculated phase and obtain the graphs for both cases shown in Figure 8.

**Figure 9:**
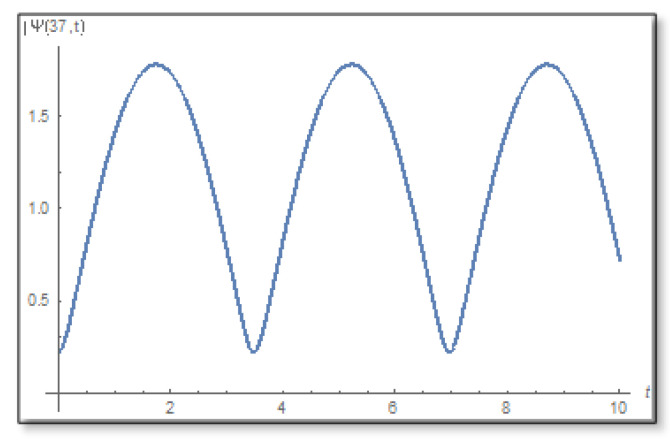
The amplitude as a function of time inside the barrier for x=37 Å for the duration 0–10. Here, too, it can be seen that the amplitude has no zeros neither on the timeline.

**Figure 10:**
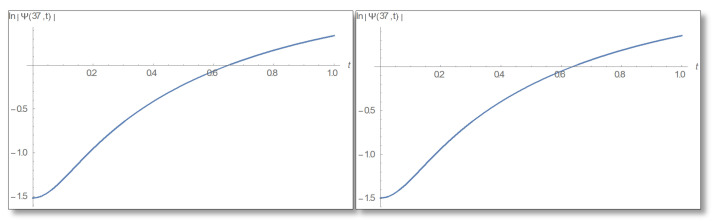
The graph of the amplitude logarithm as a function of time in the barrier for x=37 Å. On the left is the direct calculation and on the right is the calculation via the temporal Kramers–Kronig integral. It can be seen that the theory predictions are correct.

Now we study the barrier, we know that the barrier domain is certain, that is, there are no zeros at all in the complex time plane (see Figure 9). We will use the transform (56) and draw graphs in Figure 10.

**Figure 11:**
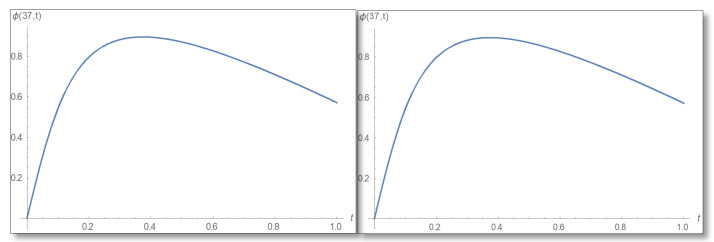
The phase as a function of time inside the barrier at point x=37 Å for the duration 0–1. On the left is the direct calculation and on the right the calculation via the temporal Kramers–Kronig integral. The graphs are identical and therefore the mathematical development is valid.

Now we turn to Equation (57) which is an inverse transform and draw a graph for the phase shown in Figure 11.

**Figure 12:**
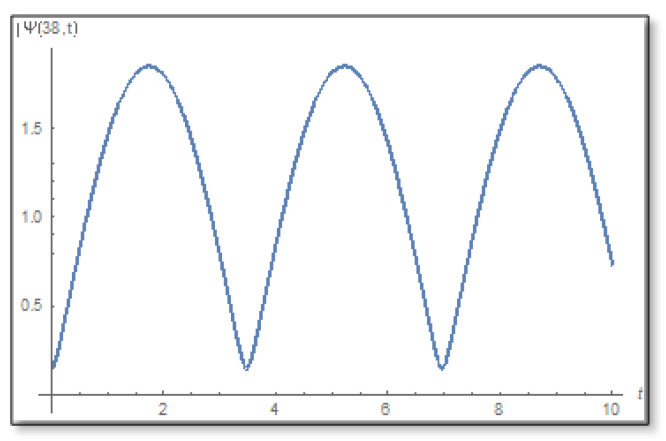
The graph of the amplitude time dependence in the third domain for x=38 Å. Here it can be seen that the amplitude has no zeros on the timeline in the duration 0–10.

**Figure 13:**
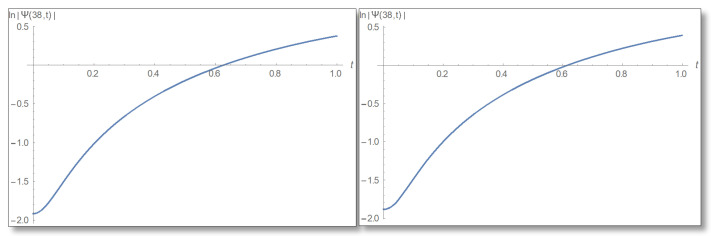
The graph of the amplitude logarithm as a time dependence in the third area for x=38 Å. On the left is the direct estimation and on the right is the calculation via the temporal Kramers–Kronig integral. The plot corroborates the theory.

Now we will turn to the third domain of the well in which the uncertainty in described by Equation (55). In this domain we choose a point close to the barrier that satisfies the conditions of certainty, for example, x=38 Å. We will refer to Equation (56) and draw graphs of the amplitude and its logarithm in Figure 12 and Figure 13.

**Figure 14:**
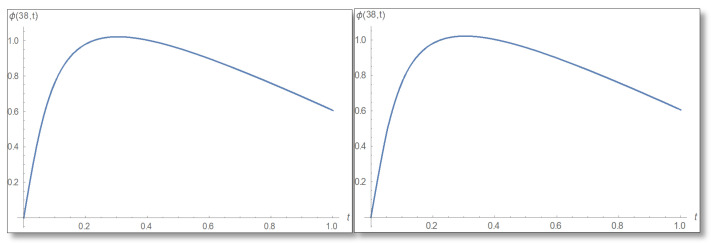
The graph of the phase as a function of time in the third domain at the point x=38 Å for the duration 0–1. On the left is the direct evaluation and on the right is the calculation via the temporal Kramers–Kronig integral. The graphs are identical and therefore the mathematical analysis is valid.

Now we turn to Equation (57) and draw a graph depicted in Figure 14.

**Figure 15:**
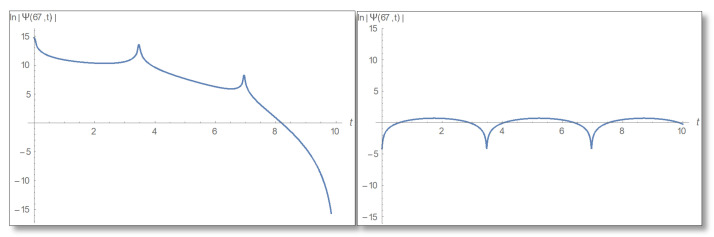
Graphs of amplitude logarithm as a function of time at point x=67 Å. The right plot is derived according to direct evaluation and left according to temporal Kramers–Kronig formal. Here it can be seen that there is no temporal Kramers–Kronig relations between phase and amplitude because at the above point there are zeros in the time plane.

From the graphs we have obtained it can be seen that indeed the relations between phase and amplitude exists according to Equations (56) and (57) but only at points where there are no zeros in the complex time plane. For completeness, we will make a temporal Kramers–Kronig evaluation at points that do not meet the conditions of certainty (points where there is no phase-amplitude relations). For example, we take x=67 Å and draw a graph of the amplitude logarithm as a function of time depicted in Figure 15.

**Figure 16:**
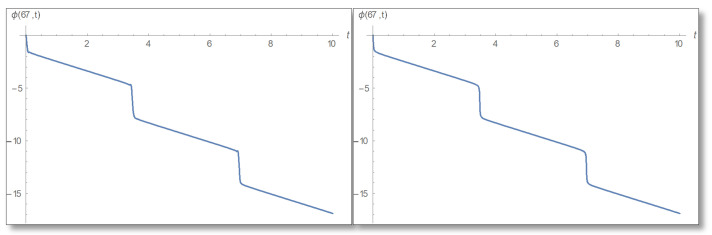
Phase as a function of time for x=67 Å. Right: Direct calculation and left temporal Kramers–Kronig evaluation. Here you see that the graphs are the same, indicating that the uncertainty relation is sufficient but not necessary for the temporal Kramers–Kronig relations to hold.

A graph of the phase as a function of time in Figure 16.

**Figure 17:**
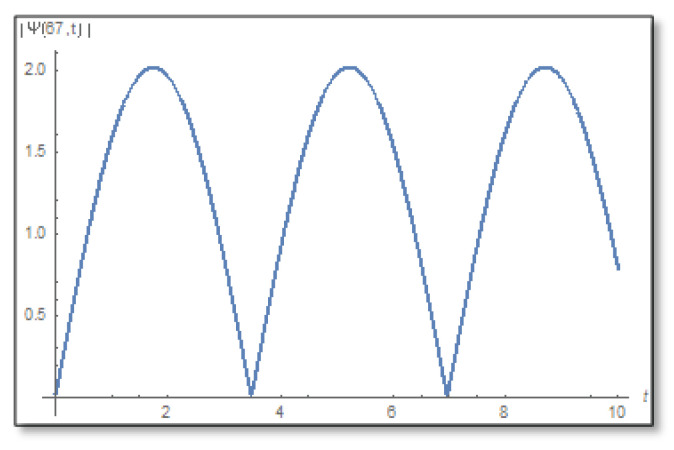
The graph of the amplitude as a function of time for x=67 Å. Here it can be seen that the amplitude has zeros on the timeline and therefore there is uncertainty.

We plot the temporal dependence of the amplitude of the wave function for x=67 Å and observe that the amplitude has zeros on the time axis and hence there is no phase-amplitude relations according to Figure 17.

## 10. General Properties of a Shannon’s Entropy in the Case of Tunneling

We shall now discuss briefly the concept of the phase of a wave function and its relationship to Shannon’s entropy associated with the wave function. Let us investigate the time evolution of the entropy [26]. We shall find it beneficial to describe the evolution of a wave function Ψ using the amplitude-phase description:

(59) $$\Psi =\left|\Psi \right|e^{\frac{i\Phi }{\hbar }},\;\phi =\frac{\Phi }{\hbar }$$

The amplitude and phase can be used to define Madelung’s density and velocity field as follows:

(60) $$\rho \equiv {\left|\Psi \right|}^{2},\;\vec{u}\equiv \frac{1}{m}\vec{\nabla }\Phi .$$

Those quantities satisfy the continuity equation:

(61) $$\partial_{t}\rho +\vec{\nabla }\cdot \left(\rho \vec{u}\right)=0,$$

and the Euler equation:

(62) $$\partial_{t}\vec{u}+\left(\vec{u}\cdot \vec{\nabla }\right)\vec{u}=-\frac{1}{m}\vec{\nabla }\left(Q+V\right),\;Q\equiv -\frac{\hbar^{2}}{2m}\frac{{\vec{\nabla }}^{2}\sqrt{\rho }}{\sqrt{\rho }}$$

in the above *Q* is the quantum potential. Those real equations are mathematically equivalent to Schrödinger’s complex equation for a single quantum particle. In terms of the probability density the entropy takes the simple form:

(63) $$S\left(t\right)=-\int_{V}\rho \;ln\;\rho \;d^{3}x$$

Taking a temporal derivative of the above quantity we obtain:

(64) $$\partial_{t}S\left(t\right)=-\int_{V}\partial_{t}\rho \left(ln\;\rho +1\right)\;d^{3}x.$$

Using the continuity Equation (61) results in:

(65) $$\partial_{t}S\left(t\right)=\int_{V}\vec{\nabla }\cdot \left(\rho \vec{u}\right)\left(ln\;\rho +1\right)\;d^{3}x.$$

The above expression can be integrated by parts and using Gauss theorem we obtain:

(66) $$\partial_{t}S\left(t\right)=\oint_{\Sigma }d\vec{A}\cdot \rho \vec{u}\left(\ln\;\rho +1\right)-\int_{V}\vec{u}\cdot \vec{\nabla }\rho d^{3}x.$$

If the surface Σ is chosen such that there is no probability flux on this surface we are left with:

(67) $$\partial_{t}S\left(t\right)=-\int_{V}\vec{u}\cdot \vec{\nabla }\rho d^{3}x=-\frac{1}{m}\int_{V}\vec{\nabla }\Phi \cdot \vec{\nabla }\rho d^{3}x=-\frac{2\hbar }{m}\int_{V}\left|\Psi \right|\vec{\nabla }\phi \cdot \vec{\nabla }\left|\Psi \right|d^{3}x.$$

Or also as:

(68) $$\partial_{t}S\left(t\right)=-\frac{2\hbar }{m}\int_{V}{\left|\Psi \right|}^{2}\vec{\nabla }\phi \cdot \vec{\nabla }\ln|\Psi |d^{3}x=-\frac{2\hbar }{m}\int_{V}\rho \vec{\nabla }\phi \cdot \vec{\nabla }\ln\left|\Psi \right|d^{3}x.$$

According to the theory of random variables the right hand side is an expectation value [27], hence:

(69) $$\partial_{t}S\left(t\right)=-\frac{2\hbar }{m}E\left[\vec{\nabla }\phi \cdot \vec{\nabla }\ln\left|\Psi \right|\right].$$

A correlation of two random variable *A* and *B* is defined as [27]:

(70) $$R_{AB}=E\left[AB\right]$$

We may generalize the above to vector quantities $\vec{A}$ and $\vec{B}$ such that:

(71) $$R_{\vec{A}\cdot \vec{B}}=E\left[\vec{A}\cdot \vec{B}\right]$$

Hence it follows that:

(72) $$\partial_{t}S\left(t\right)=-\frac{2\hbar }{m}R_{\vec{\nabla }\phi \cdot \vec{\nabla }\ln\left|\Psi \right|}.$$

Thus the entropy production rate is essentially the correlation between the gradient of phase and log amplitude of the wave function. We also notice that for independent random variables [27]:

(73) $$R_{AB}=E\left[A\right]E\left[B\right]$$

Hence, **if** one may treat $\vec{\nabla }\phi$ as independent of $\vec{\nabla }\ln\left|\Psi \right|$ we may write:

(74) $$\partial_{t}S\left(t\right)=-\frac{2\hbar }{m}E\left[\vec{\nabla }\phi \right]\cdot E\left[\vec{\nabla }\ln\left|\Psi \right|\right].$$

Now if in addition the phase is chaotic such that $E\left[\vec{\nabla }\phi \right]≃0$ the entropy production will be rather low. However, if on the other hand the temporal Kramers–Kronig relations hold those quantities are strongly correlated. It follows that one may write the entropy production rate equation using Equation (9) in the form:

(75) $$\begin{array}{ccc} \partial_{t}S\left(t\right) & = & -\frac{2\hbar }{m}E\left[\vec{\nabla }\phi \cdot \vec{\nabla }\ln\left|\Psi \right|\right]=-\frac{2\hbar }{m}E\left[\vec{\nabla }\phi \cdot \frac{1}{\pi }P\int_{-\infty }^{\infty }\frac{\vec{\nabla }\phi \left(x,t^{\prime }\right)}{t^{\prime }-t}dt^{\prime }\right]\\ & = & -\frac{2\hbar }{m\pi }P\int_{-\infty }^{\infty }dt^{\prime }\frac{1}{t^{\prime }-t}E\left[\vec{\nabla }\phi \left(\vec{x},t\right)\cdot \vec{\nabla }\phi \left(x,t^{\prime }\right)\right]. \end{array}$$

Now, according to the theory of random processes, an autocorrelation of a random process A(t) is given by [27] a correlation of two time samples of the process:

(76) $$R_{A}\left(t,t^{\prime }\right)=E\left[A\left(t\right)A\left(t^{\prime }\right)\right]$$

Thus the entropy production rate is given in terms of the phase autocorrelation as:

(77) $$\partial_{t}S\left(t\right)=-\frac{2\hbar }{m\pi }P\int_{-\infty }^{\infty }dt^{\prime }\frac{1}{t^{\prime }-t}R_{\vec{\nabla }\phi }\left(t,t^{\prime }\right).$$

We recall that autocorrelation even of white noise is not null and gives a delta function expression in time.

Now the connection between entropy increase and tunneling discussed in [26] can be also understood in the framework of the temporal Kramers–Kronig relations which are prominent in and in the vicinity of the barrier and imply correlation between phase and amplitude (as described in the above heuristic discussion). We also suggested in [26] (see also [28]) that entropy increase may be connected to the emergence of quantum chaos.

## 11. Discussion

Following Yahalom and Englman [21] who found the existence of a reciprocal relationship (temporal Kramers–Kronig) between the phase and amplitude of a wave function returned from an infinite potential barrier. Here we proved that in the case of a wave function which is a superposition of two energy eigenstates in a double potential well, temporal Kramers–Kronig relations exist in the area close to the barrier on both sides and in the barrier itself. That is, it is possible to predict the amplitude from the phase in these areas and vice versa. In the other two areas there is more information because the amplitude and phase each holds an independent amount of information.

## 12. Conclusions

We have established the connection between amplitude and phase through the temporal Kramers–Kronig relations showing that they hold only when certainty conditions are valid. It turns out that the conditions hold only in and in the vicinity of the barrier. We have also shown how phase and amplitude correlations are related to the rate of entropy increase and the development of quantum chaos.

One may claim a lack of universality of the current results. Indeed, the current analysis is limited to a specific superposition concerning two eigen-energy states. We remark, however, that in some of the cases we studied, including the present one, a type of “uncertainty relation” arises this is also correct in a wave packet colliding with an infinite potential barrier as in [21].

In a future study, it is worth examining the existence of the interrelations between phase and amplitude in very cold temperature conditions. In this case the two placement levels will be different so that the lower level of energy will be more populated. In this case it is expected that the temporal Kramers–Kronig relations will take place in a broader domain. Another direction of future research is to generalize the current results for additional energy levels.

## Data Availability

Not applicable.
